# The Lys-Asp-Tyr Triad within the Mite Allergen Der p 1 Propeptide Is a Critical Structural Element for the pH-Dependent Initiation of the Protease Maturation

**DOI:** 10.3390/ijms18051087

**Published:** 2017-05-20

**Authors:** Andy Chevigné, Vincenzo Campizi, Martyna Szpakowska, David Bourry, Marie-Eve Dumez, José C. Martins, André Matagne, Moreno Galleni, Alain Jacquet

**Affiliations:** 1 Macromolécules Biologiques, Centre for Protein Engineering, University of Liège, B-4000 Liège, Belgium; andy.chevigne@lih.lu (A.C.); vcampisi@live.be (V.C.); marie-eve.x.dumez@gsk.com (M.-E.D.); mgalleni@ulg.ac.be (M.G.); 2 Department of Infection and Immunity, Luxembourg Institute of Health (LIH), L-4354 Esch-sur-Alzette, Luxembourg; martyna.szpakowska@lih.lu; 3 NMR and Structure Analysis Unit, Department of Organic Chemistry, Ghent University, B-9000 Ghent, Belgium; David.Bourry@telenet.be (D.B.); jose.martins@ugent.be (J.C.M.); 4 Laboratoire d’Enzymologie, Centre for Protein Engineering, University of Liège, B-4000 Liège, Belgium; amatagne@ulg.ac.be; 5 Department of Medicine, Faculty of Medicine, Chulalongkorn University, Bangkok 10330, Thailand

**Keywords:** cysteine protease, Der p 1, pH sensor, pH unfolding, propeptide, maturation

## Abstract

The major house dust mite allergen, Der p 1, is a papain-like cysteine protease expressed as an inactive precursor, proDer p 1, carrying an N-terminal propeptide with a unique structure. The maturation of the zymogen into an enzymatically-active form of Der p 1 is a multistep autocatalytic process initiated under acidic conditions through conformational changes of the propeptide, leading to the loss of its inhibitory ability and its subsequent gradual cleavage. The aims of this study were to characterize the residues present in the Der p 1 propeptide involved in the initiation of the zymogen maturation process, but also to assess the impact of acidic pH on the propeptide structure, the activity of Der p 1 and the fate of the propeptide. Using various complementary enzymatic and structural approaches, we demonstrated that a structural triad K17p-D51p-Y19p within the N-terminal domain of the propeptide is essential for its stabilization and the sensing of pH changes. Particularly, the protonation of D51p under acidic conditions unfolds the propeptide through disruption of the K17p-D51p salt bridge, reduces its inhibition capacity and unmasks the buried residues K17p and Y19p constituting the first maturation cleavage site of the zymogen. Our results also evidenced that this triad acts in a cooperative manner with other propeptide pH-responsive elements, including residues E56p and E80p, to promote the propeptide unfolding and/or to facilitate its proteolysis. Furthermore, we showed that acidic conditions modify Der p 1 proteolytic specificity and confirmed that the formation of the first intermediate represents the limiting step of the in vitro Der p 1 maturation process. Altogether, our results provide new insights into the early events of the mechanism of proDer p 1 maturation and identify a unique structural triad acting as a stabilizing and a pH-sensing regulatory element.

## 1. Introduction

House dust mite (HDM) is commonly associated with allergic diseases, such as asthma, perennial rhinitis and atopic dermatitis. So far, more than 20 HDM allergens have been identified in mite feces and bodies, and several of them, including Der p 1, Der p 3 and Der p 6, have been reported to exhibit a proteolytic activity [1]. Der p 1 is a 25-kDa cysteine protease belonging to the papain-like protease family (CA1) and is a major mite allergen, as more than 80% of the HDM allergic patients develop sensitizations to this protease [2,3]. Moreover, the proteolytic activity of Der p 1 has been demonstrated to be closely related to the development of allergic diseases, notably through cleavages of tight junction proteins, immune receptors, but also the maturation of other HDM protease allergens, such as Der p 3 and Der p 6 [4,5,6,7,8,9,10,11,12]. Although the biological roles of these proteases are not well understood, they have been found in the mites’ gut and feces, suggesting their possible role in digestion [7].

Like most proteases, the secreted allergen Der p 1 is synthesized as an inactive precursor, called proDer p 1. This immature protein is composed of a catalytic domain of 222 residues preceded by an N-terminal propeptide of 80 amino acids, which not only blocks the accessibility of the protease active site, but also masks some IgE epitopes of the mature allergen [13,14]. In addition to its inhibitory properties, the propeptide was proposed to act as an intramolecular chaperone to assist the folding of the protease catalytic domain and to be involved in the addressing of the zymogen through the cell secretory pathway [7,15,16]. Considering the role and the importance of Der p 1 in HDM allergy, the elucidation of the proDer p 1 activation mechanism leading to allergen maturation through the propeptide removal is an essential step.

**Figure 1:**
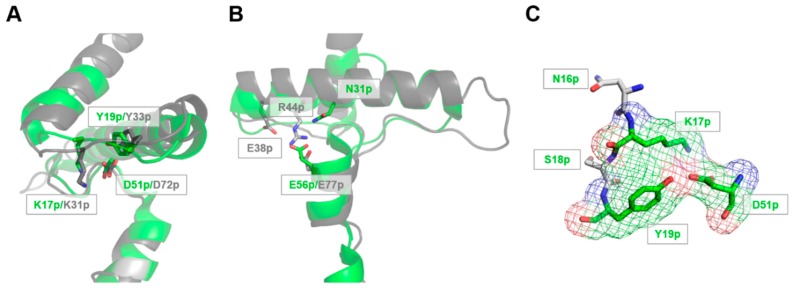
Structural comparison between the propeptides of Der p 1 and caricain. The propeptides of Der p 1 (PDB 1XKG) [22] and caricain (PDB 1PCI), a typical family L propeptide, are colored green and grey, respectively. (**A**,**B**) The overlay of the N-terminal domain shows that the Lys-Asp-Tyr (K-D-Y) triad located in the coil between α-helices 1 and 2 is conserved in both propeptides (**A**), whereas the triad composed of the Glu-Arg-Glu residues (E38pR44pFNIN motif) is not present in the propeptide Der p 1 and is replaced by a Glu-Asn (E56p-N31p) interaction (**B**); (**C**) Representation of the interactions within the Lys-Asp-Tyr triad of the Der p 1 propeptide illustrated by the electron density map.

Based on their structures, lengths and sequence similarities, propeptides of papain-like proteases are commonly classified into two distinct subfamilies [17,18]. The cathepsin L-like subfamily includes long propeptides (up to 100 residues) with three α-helices, the first two forming a globular domain, and a long C-terminal tail locking the catalytic domain, thereby preventing access to the active site. In this subfamily, the second α-helix of the N-terminal domain displays a conserved ERFNIN motif involved in the propeptide stabilization and pH sensing [19,20,21] (Figure 1).

The propeptides from the cathepsin B-like subfamily are usually shorter (60 residues), with only two α-helices and lack the ERFNIN motif. Surprisingly, the resolution of the Der p 1 zymogen X-ray structure revealed unique features within its propeptide [22]. Indeed, this prosequence, with an intermediate size of 80 residues, is composed of four α-helices with the first three forming a short N-terminal globular domain lacking the ERFNIN motif and the fourth replacing the C-terminal tail found in the two other subfamilies. Based on these characteristics, the propeptide of Der p 1 was proposed to be the first representative of a new papain-like prosequence subfamily (Figure 1) [22].

**Figure 2:**
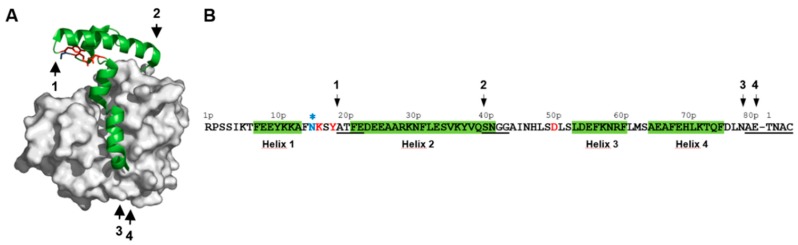
Schematic representation of Der p 1 zymogen and location of the cleavage sites identified during autocatalytic processing of proDer p 1. (**A**) Overall structure of the proDer p 1 zymogen (PDB 1XKG) [22]. The propeptide is colored green, whereas the structure of the mature protease is shown in grey; (**B**) Sequence of the Der p 1 propeptide. Helices 1, 2, 3 and 4 are represented by green boxes. The propeptide residue numbers are indicated with the letter p. Arrows 1, 2, 3 and 4 indicate the positions of the different cleavage sites generating the ATFE-, SNGG-, AETN- or TNAC-forms, respectively (underlined sequences). The residues involved in the K17p-D51p-Y19p triad are colored red. The *N*-glycosylation site corresponding to the N16p-K17p-S18p sequence located just upstream of the cleavage site generating the first intermediate (ATFE-) is colored blue and indicated by a star.

The in vitro maturation of the papain-like cysteine protease precursors commonly occurs under acidic conditions (i.e., pH 4) through progressive autocatalytic truncations of the N-terminal propeptide [23,24]. Two intermediates were identified along the maturation process of proDer p 1 and correspond to the successive loss of the first and second N-terminal α-helices of the propeptide following cleavages between residues Y19p–A20p and Q40p–S41p (p: propeptide numbering), respectively (Figure 2) [15,25,26].

The full maturation of Der p 1 is subsequently achieved after the removal of the propeptide C-terminal helix following cleavages at overlapping sites N78p–A79p or E80p–T81 generating mature protease with or without an A79p–E80p extension [25,26]. Noteworthy, several studies suggested that *N*-glycosylation of the propeptide (N16p) could impair the maturation of the allergen [7,9,27,28] (Figure 2). Although different studies clearly demonstrated that activation of papain-like zymogen under acidic conditions is related to the unfolding of the propeptide and the subsequent loss of its inhibitory capacity, the molecular determinants involved in propeptide stabilization, pH sensing, as well as the impact of acidic conditions on the specificity of the catalytic domain of cysteine proteases remain poorly investigated [15,29,30].

The aims of the present study were to investigate the early events of proDer p 1 maturation and to decipher the roles of different ionizable residues present in the propeptide responsible for its pH-dependent structural stability and inhibitory capacity. Our results demonstrated that D51p, a residue involved in a structural triad formed by residues K17p, D51p and Y19p, not only stabilizes the propeptide N-terminal domain, but also acts as the major pH-responsive element necessary for the initiation of proDer p 1 maturation.

## 2. Results

### 2.1. Identification of pH-Responsive Elements within the Der p 1 Propeptide N-Terminal Domain

The analysis of the tridimensional structure of proDer p 1 (PDB 1XKG) reveals that, together with the buried aromatic cluster involving F8p, Y11p and F32p, the structure and compactness of the propeptide N-terminal domain relies mainly on stabilizing interactions orchestrated by two different ionizable residues bearing negative charges (Figure 1) [22]. The residue D51p, located in the coil connecting the helices 2 and 3, forms a structural triad through a salt bridge with K17p and a hydrogen bond with Y19p, two residues located in the coil between the helices 1 and 2 (Figure 1A,C). The residue E56p, present in the middle of the helix 3, is hydrogen bound with the side chain of N31p located in the middle of the helix 2 (Figure 1B).

To investigate the role and contribution of these two ionizable residues and their interactions in the structure, the stability, as well as the pH-dependent inhibitory ability of the propeptide, different mutants were generated (D51pA, E56pA, D51pA/E56pA, K17pA and Y19pF) and fully characterized.

**Figure 3:**
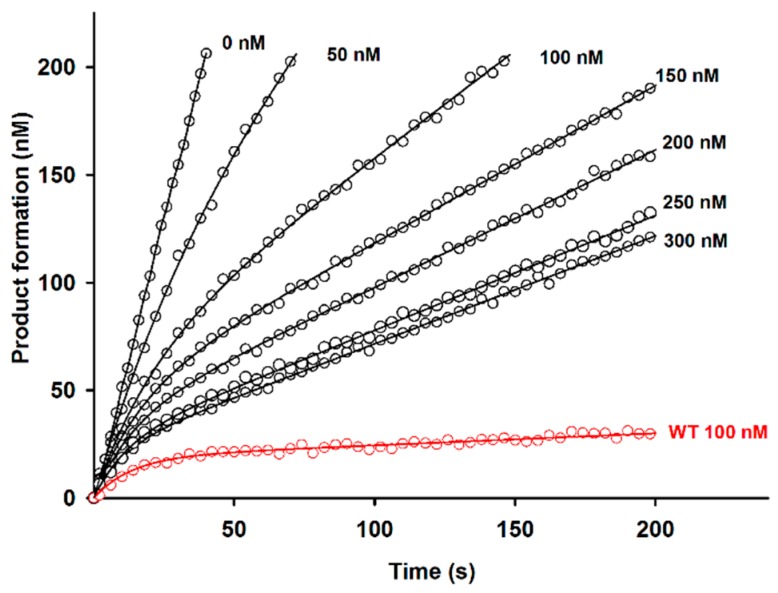
D51pA propeptides inhibitory capacity. Time-dependent inhibition of Der p 1 (5 nM) by D51pA propeptide (0–300 nM) and comparison to WT propeptide at 100 nM (red). The corresponding dissociation constant and individual association and dissociation rate constants are detailed in Table 1. The protease activity was measured at the steady-state using Boc-Gln-Ala-Arg-AMC as a substrate. The data shown are representative of three independent experiments.

Kinetic inhibition experiments revealed that, at pH 7, D51pA and K17pA mutations reduced the propeptide inhibitory capacity by five- and two-fold compared to wild-type (WT) propeptide with dissociation constants (*K*_D_) of 37 ± 5 and 15 ± 3 nM, respectively (Figure 3, Table 1). More importantly, these mutants failed to fully inhibit Der p 1 protease activity even at concentrations as high as 300 nM (Figure 3). Determination of the individual kinetic rate constants for association (*k*_on_) and dissociation (*k*_off_) showed that D51pA mutation affects both the association and the dissociation rates, whereas K17pA replacement changed only the association rate (Table 1). In contrast, mutations Y19pF and E56pA induced a slight increase of the association rates of the corresponding propeptides, but did not influence the overall affinity of the propeptide for the protease as the measured dissociation constants were similar to that of the WT propeptide. Similarly to the WT propeptide, Y19pF and E56pA mutants fully inhibited Der p 1 proteolytic activity at concentrations higher than 40 nM (Table 1). The double mutation D51pA/E56pA induced a large reduction of the propeptide inhibition ability, as illustrated by the twenty-fold increase of the *K*_D_ value (*K*_D_ = 130 ± 6 nM), suggesting that the two residues act in a cooperative manner.

### 2.2. Effect of D51pA Mutation on the pH Dependence of Propeptide Inhibition and Zymogen Activation

**Figure 4:**
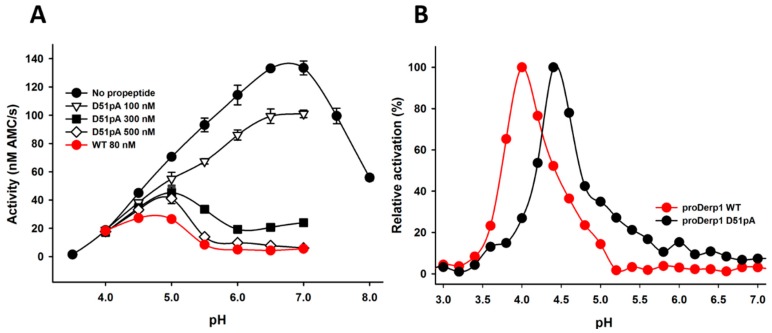
Impact of D51pA mutation on the pH-dependence of Der p 1 propeptide inhibitory capacity and proDer p 1 activation. (**A**) Inhibition of the Der p 1 protease activity by WT or D51pA propeptide monitored at different pH values. Data related to WT propeptide at 80 nM are from [15]. Inhibition is indicated as the percentage of residual activity (%) of Der p 1 considering the activity of Der p 1 in the absence of propeptide as 100%. Der p 1 protease activity was measured at the steady-state using Boc-Gln-Ala-Arg-AMC as a substrate. Data are presented as the mean of duplicates ± the standard deviation and are representative of two independent experiments; (**B**) pH dependence of WT and D51pA proDer p 1 maturation. The percentage of activation was estimated by measuring the enzymatic activity generated at pH 7 after incubation of the zymogens at pH ranging from 3–7 and considering the maximal activity obtained as 100%.

Given the major contribution of D51p in the propeptide inhibitory ability at neutral pH observed in Table 1 and Figure 3 and its role in the formation of the K17p-D51p-Y19p triad revealed by the analysis of proDer p 1 tridimensional structure (Figure 1 and Figure 2), we hypothesized that it may act as a pH sensor for the pH-dependent initiation of the protease maturation. Inhibition assays at different pH demonstrated that WT propeptide fully inhibited Der p 1 activity at pH as low as 5.5, whereas the inhibitory capacity of the D51pA mutant was largely reduced at pH 6 and abolished at pH 5.5 (Figure 4A).

To further evaluate the contribution of D51p in the processing of proDer p 1, the maturation profile of unglycosylated WT and D51pA proDer p 1 was compared at different pH (Figure 4B). Whereas the maturation of unglycosylated wild-type zymogen was restricted to pH values ranging from 3.5–5, with an optimal activation at pH 4, the D51pA mutant displayed a shift of the optimal pH, ranging from pH 4–6. However, its maturation remained clearly more efficient at acidic pH.

### 2.3. Impact of Mutations on the Propeptide Proteolytic Degradation by Der p 1

**Figure 5:**
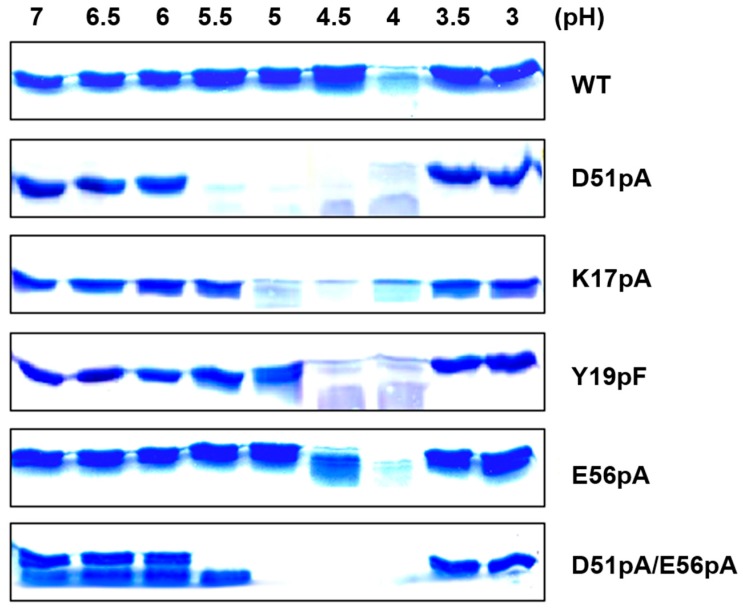
Impact of mutation on the susceptibility to Der p 1 proteolysis of the propeptide variants at different pH. WT, as well as D51pA, K17pA, Y19pF, E56pA, D51pA/E56pA propeptide mutants were incubated with recombinant Der p 1 at different pH values, and proteolysis was detected by SDS PAGE stained by Coomassie blue. M: molecular weight (10 kDa). Data shown are representative of two independent experiments.

To evaluate the contribution of the K17p–D51p–Y19p triad and E56p residue in the pH dependence of the propeptide compactness and, consequently, sensitivity to proteolysis, the different propeptide variants were treated with Der p 1 at different pH values, and the degradation patterns were analyzed by SDS-PAGE (Figure 5). Proteolysis of WT propeptide was restricted to pH 4, the absence of propeptide degradation at lower pH values being due to the inactivation of the Der p 1 protease [15]. The proteolysis of the D51pA and K17pA mutants was already observed at pH 5.5 and 5, respectively, confirming that the disruption of the salt bridge between K17p and D51p induces the relaxation of the N-terminal domain and that D51pA mutation has a higher impact than K17pA mutation. For both Y19pF and E56pA mutants, proteolysis was observed at pH 4 and 4.5. The degradation profile of the D51pA/E56pA double mutant was similar to that observed with the D51pA mutant. However, a slight degradation was already detected at pH 7 for this mutant, confirming that the two ionizable groups could both contribute to the propeptide stability.

### 2.4. Impact of Mutations on the Tertiary and Secondary Structures of the Propeptide

**Figure 6:**
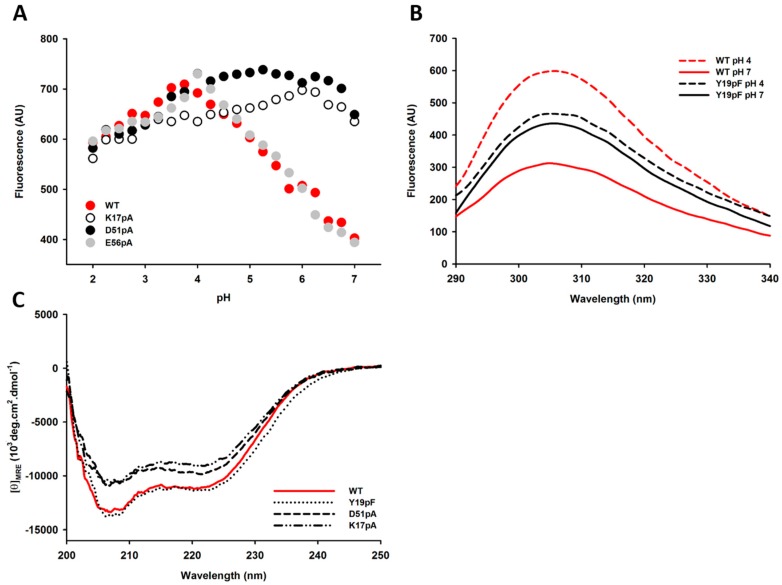
Structural analysis of the propeptide mutants and pH dependence of the conformational changes. (**A**) Effect of pH on intrinsic tyrosine emission fluorescence intensity at 305 nm for WT, D51pA, K17pA and E56pA propeptides; (**B**) intrinsic emission fluorescence spectra of WT and Y19pF mutant at pH 7 and 4; (**C**) UV-Circular dichroism spectrum of WT, D51pA, K17pA, Y19pF mutants at pH 7. Data shown are representative of two independent experiments.

In the absence of tryptophan residues, the intrinsic fluorescence of the Der p 1 propeptide largely depends on the solvent accessibility and the protonation state of three tyrosine residues (Y11p, Y19p and Y38p) buried in the propeptide N-terminal domain [15,22]. Measurements of the fluorescence emission intensity revealed that the WT and E56pA propeptides share a similar tyrosine fluorescence profile that increases with decreasing pH values to reach a maximum at pH 4 (Figure 6A). In contrast, the intrinsic fluorescence intensities of D51pA and K17pA mutants were not influenced by pH changes and were already maximal at pH 7, indicating that the tyrosine residues from these two mutants are already largely accessible to the solvent and/or fully protonated at neutral pH. Interestingly, no change in the intrinsic fluorescence intensity was observed for the Y19pF mutant between pH 7 and 4, although this mutant displays inhibition properties equivalent to the WT propeptide (Figure 6B).

Far-UV circular dichroism (CD) spectra measured at pH 7 evidenced that, compared with the WT and Y19pF propeptides, K17pA and D51pA mutants displayed a slight decrease in their α-helical content (Figure 6C) similar to the reduction observed for WT propeptide at pH 4 [15].

**Figure 7:**
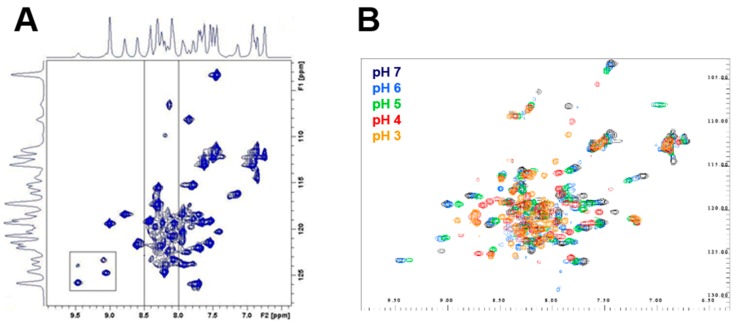
NMR spectra and pH titration of WT propeptide N-terminal domain. (**A**) 2D ^1^H–^15^N HSQC of WT propeptide N-terminal domain (R1–R60) at pH 7 and 20 °C. The lined box indicates areas suggestive of the presence of multiple conformations for the propeptide in solution. The area between 8 and 8.5 ppm is defined by vertical lines; (**B**) pH titration of WT propeptide by 2D ^1^H–^15^N in the amide region at pH 7 (black), pH 6 (blue), pH 5 (green), pH 4 (red) and pH 3 (orange) at 20 °C.

NMR spectroscopy and hydrogen/deuterium exchange experiments were performed to further characterize the conformational changes of the propeptide N-terminal domain under acidic conditions. As highly concentrated soluble propeptide is needed for NMR experiments, the hydrophobic fourth α-helix was deleted to produce only the propeptide N-terminal domains (R1p–R60p). The 1D ^1^H and 2D NOESY spectra of the WT propeptide measured at pH 7 indicated the presence of a folded conformation, as judged from the overall spread of the ^1^H resonances (Figure 7A). These peaks provided a useful fingerprint of the propeptide structure, which was subsequently used to investigate the pH unfolding of the WT propeptide and the effect of the D51pA mutation on the propeptide structural flexibility.

### 2.5. Differential Flexibility and Compactness of WT and D51pA Propeptides

**Figure 8:**
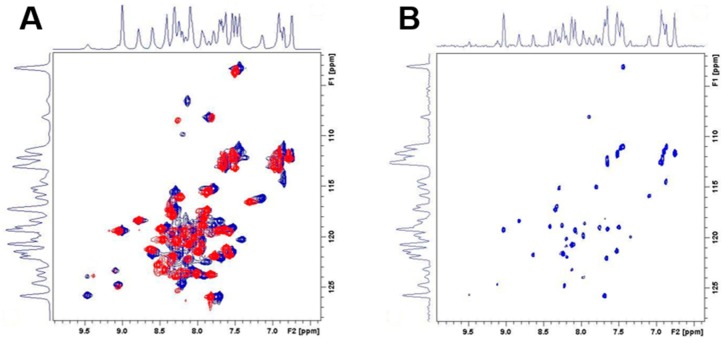
Hydrogen/deuterium (H/D) exchange analysis of WT and D51pA proDer p 1 monitored by NMR. (**A**) Overlay of the WT (blue) and D51pA (red) proDer p 1 2D ^1^H–^15^N HSQC spectra at pH 7 and 20 °C; (**B**) 2D ^1^H–^15^N HSQC spectrum showing the fingerprint of WT proDer p 1 90 min after H/D exchange. All of the peaks of D51pA propeptide vanished, demonstrating the high flexibility of its N-terminal domain.

2D ^1^H–^15^N HSQC spectra of the WT propeptide N-terminal domain recorded at every pH unit identified some broad resonances peaks slightly moving from pH 7–5, followed by a more drastic transition between pH 5 and 4. These observations likely reflect a progressive conformational change of the propeptide N-terminal domain, which accelerates from pH 5 affecting the complete protein fingerprint (Figure 7B). These gradual changes were followed by an even more rapid and drastic shifts between pH 4 and 3, characteristic of a loss of the tertiary and secondary structure. Similar NMR patterns were observed for the D51pA propeptide at pH 7 (Figure 8A) confirming that this mutation did not perturb the overall fold of the propeptide, which correlates with the maintenance, albeit reduced, of the inhibitory ability of this propeptide variant (*K*_D_ = 37 ± 5 nM) and of its overall secondary structure.

To further investigate the impact of D51pA mutation on the kinetic stability and compactness of the propeptide N-terminal domain, H/D exchange experiments using 2D ^1^H–^15^N HSQC spectra were performed. For the WT N-terminal domain, an important fraction of amide cross-peaks vanished within 90 min (Figure 8B). Nevertheless, a considerable number of peaks remained visible, indicating that a fraction of the amides is involved in hydrogen bonds or shielded from the solvent, most probably tightly buried in the N-terminal domain. In contrast, the 2D ^1^H–^15^N HSQC spectrum of the D51pA mutant at the same time point was empty. This marked difference indicated a decrease in the kinetic stability of the fold at pH 7 caused by the D51pA mutation (Figure 8B). This observation demonstrates that, although the overall propeptide structure is conserved in the D51pA mutant, the disruption of the triad greatly increases the flexibility of the propeptide N-terminal domain, rendering the otherwise hidden residues more accessible to solvent.

### 2.6. Impact of pH on proDer p 1 Activation Site Recognition and Proteolysis

The impact of pH on the recognition and cleavage efficacy of the different proDer p 1 activation sites (NKSY_19p_–A_20p_TFE, KYVQ_40p_–S_41p_NGG, FDLN_78p_–A_79p_ETN and LNAE_80p_–T_1_NAC) was also evaluated using the corresponding FRET substrates treated with Der p 1 (Figure 2).

At pH 7, Der p 1 displayed contrasting catalytic efficiency values (*k*_cat_/*K*_m_) towards the different substrates (Table 2).

**Figure 9:**
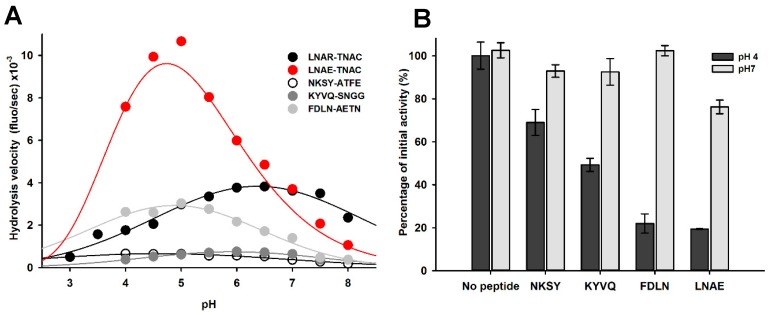
Interaction of Der p 1 with peptides covering the sequences of the different zymogen activation sites. (**A**) Effect of pH on the hydrolysis of octapeptidyl FRET substrates (2.5 µM) corresponding to proDer p 1 maturation sites by Der p 1 (5 nM); (**B**) Inhibitory activity of tetrapeptides (10 mM) mimicking the activation sites. All activity data are expressed as the mean ± standard error from at least two independent experiments performed in duplicate.

LNAE_80_–T_1_NAC was the most efficiently cleaved substrate followed by FDLN_78p_–A_79p_ETN, KYVQ_40p_–S_41p_NGG and NKSY_19p_–A_20p_TFE. Such efficiency perfectly matched the reverse order of appearance of the corresponding intermediates species during the proDer p 1 maturation process (Figure 2). This suggests that the recognition and the cleavage of the first activation site (NKSY_19p_–A_20p_TFE) is the limiting step of the zymogen proteolysis process. Attempts to determine the catalytic efficiency values (*k*_cat_/*K*_m_) at pH 4 were carried out but failed to provide robust and significant data due to the reduced activity of Der p 1 at this pH and substrate inhibition phenomena observed for LNAE_80p_–T_1_NAC, FDLN_78p_–A_79p_ETN and NKSY_19_–A_20_TFE at concentrations higher than 5 µM . Nevertheless, to assess the impact of pH on the recognition and cleavage of the different maturation sites, the hydrolysis of the different substrates was monitored and compared at pH from 8–4 at a unique substrate concentration of 2.5 µM for which no substrate inhibition was observed (Figure 9A). The hydrolysis of NKSY_19p_–A_20p_TFE and KYVQ_40p_–S_41p_NGG was weak compared to the three other peptides. The cleavage of FDLN_78p_–A_79p_ETN was slightly fostered at pH from 6.5–4.5. The most striking observation was made with LNAE_80p_–T_1_NAC, whose cleavage was largely enhanced under acidic conditions; from pH 4.5–5.5 compared to LNAR_80p_–T_1_NAC. These data suggest that the protonation of the carboxylic group of E80p side chain, and most probably that of D76p, are critical for efficient recognition and cleavage of the peptide by Der p 1.

As a complementary approach, the recognition of the propeptide cleavage sites was further investigated using synthetic tetrapeptides corresponding to the first half of the four cleavage sites identified during activation (NKSY_19p_, KYVQ_40p_, FDLN_78p_ and LNAE_80p_) by assessing their ability to compete with the QAR-AMC substrate for the Der p 1 catalytic site at pH 7 and 4 (Figure 9B). At neutral pH, only LNAE peptide was able to inhibit partially the protease activity, confirming the results observed with FRET substrates (Figure 9B). In contrast, although the enzyme was less active at pH 4, the competition assay evidenced a reduction of 30%, 45%, 75% and 80% of the initial activity with the peptides NKSY, KYVQ, FDLN and LNAE, respectively.

## 3. Discussion

Maturation of papain-like (CA1) proteases results from the loss of the N-terminal inhibitory propeptide, leading to the release of the catalytic activity tightly influenced by environmental changes such as pH changes or interaction with glycosaminoglycan [24,31,32,33]. For CA1 proteases, the autocatalytic maturation process occurs under acidic conditions following partial pH-dependent unfolding of the propeptide, which releases sufficient proteolytic activity to initiate the cleavage of the propeptide. The unfolded propeptide becomes thereby a substrate for the catalytic domain and is degraded through intra- and/or inter-molecular cleavages. Therefore, the ability to sense environmental pH changes and propeptide unfolding are the two critical steps for the initiation of the cysteine protease maturation. Tight regulation of these steps could prevent uncontrolled activation leading to damages of the producing cells under physiological conditions.

### 3.1. The Lys17p-Asp51p-Tyr19p Triad Is the Major Structural pH-Responsive Element in Der p 1 Propeptide

Structural analysis of Der p 1 propeptide revealed the residues D51p and E56p as two potential major pH sensors for the initiation of the propeptide unfolding under acidic conditions. Indeed, in addition to their key role in stabilizing salt bridge and/or hydrogen bonds responsible for the appropriate orientation and compactness of the three α-helices, these two acidic residues are also solvent-exposed to sense environmental pH changes. Through different experimental approaches, this study showed that the K17p–D51p salt bridge represents the most important stabilizing interaction of the propeptide N-terminal domain. Indeed, significant decreases in the propeptide affinity (*K*_D_) and important increases in the N-terminal domain flexibility and proteolysis sensitivity were observed for mutants D51pA and K17pA (Figure 3 and Figure 6, Table 1). By contrast, mutation E56pA had almost no effect on the propeptide structure and inhibitory ability. However, when D56pA and D51pA were combined, the propeptide displayed an affinity reduced by more than three-fold compared to that of the D51pA mutant alone and twenty-fold compared to WT propeptide. Moreover, D51pA/E56pA propeptide was shown to be much more sensitive to proteolysis at neutral pH than the D51pA mutant, pointing to a synergistic effect of the two mutations. Although E56p is not implicated in a typical triad, like the E-R-E triad characteristic of the cathepsin L-subfamily, the cooperative effect observed with the D51pA–E56pA mutant may be explained by the implication of E56p in a hydrogen bond with the side chain of the N31p located in the middle of the helix 2. This interaction may play a similar role as the E-R-E triad by orientating the helix 3 towards the active site of the catalytic domain (Figure 1B) [20].

The modest impact of Y19pF mutation on the propeptide affinity and proteolysis sensitivity suggests that the hydrogen bond between D51p and Y19p does not greatly contribute to the stabilization of the propeptide structure. However, the D51pA mutation, leading to the loss of both the salt bridge with K17p and the hydrogen bond with Y19p, drastically reduces the propeptide affinity compared with the K17pA substitution, suggesting that these two stabilizing interactions may have synergistic effect within the triad. Compared with the WT propeptide, the Y19pF mutant displayed similar inhibitory properties, but no change in the intrinsic fluorescence intensity under acidic conditions. Therefore, the pH-dependence of the propeptide fluorescence intensity likely reflects changes in the solvent accessibility of Y19p. This residue, buried within the propeptide structure at pH 7, becomes progressively exposed to the solvent at acidic pH when its interactions with gradually protonated D51p are altered. This disruption of the triad may thus increase the propeptide N-terminal domain flexibility and, consequently, potentiate its proteolysis sensitivity as evidenced by D51pA, K17pA and D51pA/E56pA mutants.

### 3.2. The Release and Docking of the Asn16p-Lys17p-Ser18p-Tyr19p Sequence Regulates the Activation Process

Previous in vitro studies showed that the pH-induced proDer p 1 maturation is a multi-step process initiated with the cleavage of the peptide bond between Y19p–A20p generating the so-called A_20p_TFE-intermediate [15,25,26]. This cleavage occurs within the solvent-exposed coil connecting the first two α-helices of the propeptide, directly downstream of the N16p–K17p–S18p–Y19p sequence. For efficient cleavage and release, the side chains of this propeptide segment need to be accessible for appropriate docking into the Der p 1 substrate specificity pockets. Noteworthy, residues K17p and Y19p, which are present at the first cleavage site, are also part of the K17p–D51p–Y19p triad and are thus buried inside the N-terminal domain of the propeptide at neutral pH. Our results demonstrated that under acidic conditions, protonation of D51p disrupts the triad. This rearrangement leads to an increase of the propeptide N-terminal domain flexibility and finally enhances the solvent accessibility of the K17p and Y19p side chains, thereby facilitating the formation of the first activation intermediate displaying the N-terminus sequence ATFE (A_20p_TFE).

The formation of the A_20p_TFE-intermediate has previously been proposed to be a major limiting step of the activation process as a truncated propeptide lacking the first α-helix (A20p–E80p) was shown to be devoid of inhibitory ability [15]. In line with this assumption, the enzymatic analysis of FRET substrate hydrolysis highlighted that the NKSY_19_–A_20_TFE sequence is, among the four activation sites, the least efficiently cleaved by Der p 1 at both neutral and acidic pH. Moreover, N glycosylation at the adjacent N-X-S/T site (N16p–K17p–S18p) likely accounts for the poor cleavage susceptibility through steric hindrance. It must be pointed out that proDer p 1 glycan structures generated by the yeast *Pichia pastoris* have previously been shown to decelerate the protease maturation [28]. Although the propeptide glycosylation at this site was not evidenced in the proDer p 1 naturally produced in the mite, similar “glycosylation lock” has been reported for the recombinant form of another HDM protease zymogen, proDer p 3. In this zymogen, glycosylation at the N9p, located three residues upstream of the unique activation site, has also been shown to reduce the intermolecular activation by Der p 1 [7,9].

Finally, besides propeptide unfolding, our data demonstrated that acidic pH also modifies Der p 1 substrate specificity. The recognition and processing are more efficient at low pH for peptides having a negatively-charged side chain at position P1, likely through neutralization of the negative charge. This observation is consistent with the marked preference of Der p 1 for positive or neutral amino acids in P1 at pH 7 [34]. It should be pointed out that we have previously highlighted the impact of low pH on the unfolding of Der p 1 catalytic domain and the charges decorating the propeptide binding groove [35]. Taken together, these data give strong evidence that acidic pH drastically alters the propeptide structure and activity, but also affects the catalytic domain of Der p 1 finely regulating the zymogen activation mechanism. Although Der p 1 propeptide appears to differ structurally from propeptides of the classical cathepsin L and B families, the Tyr-Asp-Lys triad is highly conserved among the members of the family L and may therefore play a similar important role in pH sensing and propeptide unfolding.

## 4. Materials and Methods

### 4.1. Expression of Der p 1 Propeptide Variants

The cDNAs encoding N-terminally His-tagged full-length (1–80) Der p 1 propeptide mutants K17pA, Y19pF, D51pA, E56pA and D51pA/E56pA, as well as the C-truncated (1–60) D51pA form were amplified by overlapping PCR using the expression vector pET-22b-Propeptide as template [15]. The PCR products were cloned into pGEM-T Easy (Promega, Madison, USA) and sequenced. The different propeptide coding cassettes were digested with *Nde*I and *Xho*I and cloned into the pET-22b(+) (Merck, Darmstadt, Germany), previously digested with the same enzymes. *Escherichia coli* BL21 (DE3) cells (Merck) were transformed by the respective expression vectors. The expression and purification of the propeptide variants were performed as previously described [15]. Their purity was assessed by SDS-PAGE, N-terminal sequencing and mass spectrometry, and their concentration was determined using the BCA assay (Thermo Fischer, Waltham, MA, USA) and bovine serum albumin (BSA) as the standard.

### 4.2. Expression and Maturation of proDer p 1 Variants

The cDNAs encoding the C-terminally His-tagged unglycosylated (N16pQ/N52Q) proDer p 1 and the unglycosylated D51pA mutant were amplified by overlapping PCR and cloned in pPIC9K expression vectors (Invitrogen, Carlsbad, CA, USA) as previously described [15]. The *P. pastoris* SMD1168 strain (Invitrogen) was transformed with the pPIC9K vectors by electroporation. Transformants were first selected for (His+) auxotrophy followed by increasing geneticin (G418) (Invitrogen) resistance (0.25–3 mg/mL). The selected clones were grown at 30 °C in 100 mL of yeast glycerol buffered media (BMGY) until an *A*_600_ value of 2–6 was reached. This culture was then transferred into 3.5 litters of BMGY and grown for 24 h at 30 °C pH 6. Production of the recombinant proDer p 1 was then induced by addition of methanol over four days (final concentration 0.5%). The methanol feed rate was regulated by monitoring the dissolved oxygen level (30%). The supernatant was finally recovered by centrifugation of the culture at 13,000× *g* for 20 min.

For the autocatalytic maturation, the corresponding recombinant yeast culture supernatants were first concentrated ten times by ultrafiltration (cut off: 10 kDa) (Merck). The concentrated zymogens (50 µL) were then diluted seven times with 50 mM polybuffer pH 2–8 (Tris, phosphate, citrate, acetate and KCl, adjusted to chosen pH with HCl) [15] containing 1 mM DTT and 1 mM EDTA and incubated for 1 h at 37 °C. The activity of the matured Der p 1 variants, diluted 10 times in 50 mM polybuffer pH 7 containing 1 mM DTT and 1 mM EDTA, was monitored at 405 nm in a PowerWave X spectrophotometer (Bio-Tek instruments, Winooski, VT, USA) using Boc(*N*-*t*-butoxycarbonyl)-Gln-Ala-Arg-pNA (paranitroanilide) (500 µM) as the substrate (Bachem, Budendorf, Switzerland).

### 4.3. Propeptide Degradation Assay

The proteolytic degradation of the propeptide variants by recombinant active WT Der p 1 was monitored at 37 °C and using a 1/50 enzyme/propeptide ratio. To assess the pH dependence of the proteolysis, experiments were performed at different pH values (pH 3.5–7) at 0.5 pH unit intervals, in 200 µL of 50 mM polybuffer containing 1 mM DTT and 1 mM EDTA and then analyzed by SDS-PAGE 18% and stained with Coomassie blue.

### 4.4. Inhibition of the Recombinant Der p 1 Enzymatic Activity by Propeptide Variants

The dissociation constants between the propeptide variants and recombinant Der p 1 were determined as previously described and considering a simple 1/1 interaction stoichiometry [15]. The proteolytic activity of Der p 1 (5 nM) was monitored in the presence or in the absence of various propeptide concentrations (30–300 nM), with a LS 50 B fluorimeter (Perkin Elmer, Wellesey, MA, USA) and using the fluorogenic substrate Boc-Gln-Ala-Arg-AMC (7-amino-4-methylcoumarin); (Bachem, Budendorf, Switzerland) [15]. For each kinetic measurement, pre-steady-state analysis allowed the determination of the pseudo-first order rate constant (*k*_obs_), characteristic of the formation of the complex, by fitting the decrease in the initial rate of the reaction (*v*_0_) to its steady-state rate (*v*_s_), according to Equation (1). *k*_obs_ depends on propeptide concentration ([*I*]) according to Equation (2). The dissociation constant (*K*_D Global_) and the individual kinetic rate constants for association (*k*_on_) and dissociation (*k*_off_) were then determined by linear regression of the first order rate constant (*k*_obs_) as a function of the propeptide concentration, according to Equations (2) and (3):

(1) $$p=v_{s}t+\left(v_{0}-v_{s}\right)\frac{\left(1-e^{-k_{\mathrm{obs}}t}\right)}{k_{\mathrm{obs}}}$$

(2) $$k_{\mathrm{obs}}=k_{\mathrm{off}}\left(1+\frac{\left[I\right]\;K_{m}}{K_{\mathrm{DGlobal}}\;(K_{m}+\left[S\right])}\right)$$

(3) $$K_{\mathrm{DGlobal}}=\frac{k_{\mathrm{off}}}{k_{\mathrm{on}}}$$

For the D51pA/E56pA mutant, the dissociation constant was calculated using Equation (4) due to the small differences between the initial (*v*_0_) and steady-state (*v*_s_) velocities. In this equation, *V*_m_ is the maximal velocity with a substrate concentration [*S*] of 160 µM and *v_i_* the steady-state velocity recorded for each propeptide concentration [*I*].

(4) $$\frac{V_{m}}{v_{i}}=1+\frac{K_{m}}{\left[S\right]}+\frac{\left[I\right]\;K_{m}}{K_{D}\;\left[S\right]}$$

### 4.5. Proteolytic Activity of Der p 1 towards proDer p 1 Activation Site-Based FRET Substrates and Inhibition of Der p 1 Activity by Tetrapeptides Mimicking the Intermediate Processing Sites

FRET (Förster Resonance Energy Transfer)-based octapeptides (NKSY_19p_–A_20p_TFE, KYVQ_40p_–S_41p_NGG, FDLN_78p_–A_79p_ETN, LNAE_80p_–T_1p_NAC) corresponding to the different Der p 1 cleavage sites of the WT propeptide were purchased from JPT (Berlin, Germany) with an N-terminal 4-(4-dimethylaminophenylazo)benzoic acid group (Dabcyl) as the quencher and a C-terminal glutamate-linked 5-((2-aminoethyl)amino)naphthalene-1-sulfonic acid (EDANS) group as the fluorophore. Tetrapeptides NKSY, KYVQ, FDLN and LNAE mimicking the successive cleaved activation sites were prepared by solid-phase peptide synthesis strategy on a PS3 automated peptide synthesizer (Protein Technologies, Inc., Tucson, AZ, USA) using N-α fluorenylmethoxycarbonyl (Fmoc)-based chemistry on Wang resin. The purity of the different peptides and their sequences were characterized by mass spectrometry and NMR. The proteolytic activity of Der p 1 towards these substrates was monitored by following the fluorescence emission increase resulting from FRET with excitation and emission wavelengths of 336 and 490 nm, respectively; using an LS 50 B fluorimeter (Perkin Elmer, Wellesey, MA, USA). The proteolytic activity of Der p 1 in the presence of tetrapeptides was monitored by following the fluorescence emission increase resulting from the hydrolysis of the substrates Boc-Gln-Ala-Arg-AMC. All experiments were carried out at 25 °C in 100 µL stirred cell, in 50 mM polybuffer adjusted to the desired pH and containing 1 mM DTT and 1 mM EDTA. For all FRET substrates, the measured EDANS fluorescence intensity was normalized taking the total fluorescence value after complete substrate hydrolysis into account.

### 4.6. Intrinsic Fluorescence Measurements of Der p 1 Propeptide

The intrinsic fluorescence emission spectra of the propeptide variants were recorded at 25 °C in 50 mM polybuffer, pH 2–9, using a scan speed of 280 nm per minute and a 5-µM protein concentration. Intrinsic fluorescence emission spectra were recorded from 285–400 nm with an excitation wavelength of 280 nm (LS 50 B fluorimeter, Perkin Elmer, Wellesey, MA, USA). Each spectrum was recorded four times, averaged and corrected for background buffer fluorescence.

### 4.7. Far-UV Circular Dichroism Spectroscopy

Far-UV CD spectra (200–250 nm) were recorded with a Jasco J-810 spectropolarimeter (Easton, PA, USA) at 20 °C in Milli-Q water, using a 1-mm path length quartz Suprasil cell (Hellma, Müllheim, Germany), with protein concentrations of ca. 0.1 mg/mL. Five scans (20 nm/min, 1-nm bandwidth, 0.2-nm data pitch and 2-s data integration time) were averaged; base lines were subtracted; and no smoothing was applied. Data are presented as the residue ellipticity ([*θ*]_MRW_) calculated using the molar concentration of protein and number of residue.

### 4.8. Nuclear Magnetic Resonance Spectroscopy

WT and D51pA propeptides were produced with a deletion of the fourth α-helix (1–60) in order to enhance the solubility at high concentrations and to focus on the N-terminal domain. The C-terminal truncated ^15^N labelled propeptides were prepared by growing bacteria in M9 minimal media supplemented with 1 g/L of ^15^N NH_4_Cl (CIL, Andover, MA, USA), and the expression was induced for 4 h with IPTG 1 mM. The exact molecular mass and the percentage of ^15^N nitrogen incorporation determined by ESI-MS analysis were 8005 Da/99% and 7962 Da/99% for the WT and D51pA propeptides, respectively. Samples for NMR analysis typically consisted of 1 mM solutions of purified ^15^N-enriched propeptide in a phosphate buffer at pH 7 with 90/10 H_2_O/D_2_O. Hydrogen/deuterium exchange data were recorded on samples prepared by adding D_2_O to the lyophilized, hydrogenated C-truncated propeptides. The absence of deleterious impact of lyophilization on the protein was confirmed as the 2D ^1^H–^15^N HSQC spectrum of a sample measured in the aforementioned buffer before and after the lyophilization process remained unchanged. All NMR measurements were performed on a Bruker AVANCE II spectrometer (Billerica, MA, USA) operating at a respective ^1^H and ^15^N frequency of 700.13 and 70.94 MHz and equipped with a ^1^H, ^13^C, ^15^N TXI-Z probe. The sample temperature was set to 30 °C, unless otherwise mentioned. 1D spectra were typically recorded with 128 scans using presaturation. All 2D spectra were typically recorded using standard pulse sequences as present in the Bruker library. The NOE mixing time was 300 ms. Typically, 2048 data points were sampled for 64 scans in the direct dimension with 512 data points in the indirect dimension and the ^1^H spectral width set to 16 and 40 ppm along the ^1^H and ^15^N dimension, respectively. For the gradient selected 2D ^1^H–^15^N HSQC, 64 scans were recorded. For 2D processing, the spectra were zero filled up to a 4096 × 2048 data matrix. Before Fourier transformation, all spectra were multiplied by a squared cosine bell function in both dimensions. All processing was performed using Topspin 2.1 and Amix 3.7.10 (Bruker).

## 5. Conclusions

In conclusion, our study provides new insights into the early events that regulate the initiation of the Der p 1 maturation. It sheds light on how changes in the environmental pH are detected and used to induce propeptide unfolding and subsequent proteolysis. The pH-dependent molecular mechanism that we propose should be correlated with the findings that Der p 1 is secreted by the epithelial cells lining the anterior midgut of the mite, a region with an acidic environment (pH 4) [36,37]. In addition to allowing a better understanding of the activation mechanism of Der p 1, our results pave the way for future applications, including optimized production of fully-enzymatically-active recombinant Der p 1 and the design of specific inhibitors for the treatment of HDM-allergic patients.

## Figures and Tables

**Table 1:** Affinity and association/dissociation rate constants of propeptide variants at pH 7.

Constants	WT	D51pA	K17pA	Y19pF	E56pA	D51pA/E56pA
*k*_on_ (M^−1^·s^−1^ × 10^4^)	110 ± 2	44 ± 7	46 ± 4	120 ± 4	150 ± 5	ND
*k*_off_ (s^−1^ × 10^−3^)	7.8 ± 1	17 ± 2	7 ± 1	7 ± 2	7 ± 3	ND
*K*_D Global_ (M × 10^−9^)	7 ± 1	37 ± 5	15 ± 3	6 ± 2	5 ± 2	130 ± 6

ND: Not determined, WT: wild-type. Data show mean affinity and rate constants + standard error of the mean of three independent experiments.

**Table 2:** Hydrolysis of FRET substrates corresponding to proDer p 1 maturation sites by Der p 1.

Cleavage Site	FRET Substrate	*k*_cat_/*K*_m_ (min^−1^·mM^−1^) *
NKSY_19p_–A_20p_TFE	*Dabcyl*-NKSY↓ATFE-*EDANS*	1221.7 ± 142.3
KYVQ_40p_–S_41p_NGG	*Dabcyl*-KYVQ↓SNGG-*EDANS*	2722.4 ± 119.7
FDLN_78p_–A_79p_ETN	*Dabcyl*-FDLN↓AETN-*EDANS*	3863.0 ± 722.0
LNAE_80p_–T_1_NAC	*Dabcyl*-LNAE↓TNAC-*EDANS*	6614.2 ± 204.0

* pH 7, ↓ indicates the cleavage site. Data show mean specific activity of recombinant Der p 1 towards the different FRET substrates + SEM and are representative of two independent experiments.
